# Digital Imaging of Peripheral Blood Smear With MC‐80 as a Screening Tool for Thalassemia

**DOI:** 10.1002/jcla.25135

**Published:** 2024-12-15

**Authors:** Peempol Chokchaipermpoonphol, Satana Lamtanthong, Sathaporn Nokkaew

**Affiliations:** ^1^ Department of Pathology, Faculty of Medicine Prince of Songkla University Songkhla Thailand

**Keywords:** diagnostic tools, digital microscopy, peripheral blood smear, screening, thalassemia

## Abstract

**Background:**

Thalassemia, a genetic blood disorder, poses significant global health challenges, emphasizing the importance of accurate screening methods. Traditional diagnostic tools, such as osmotic fragility and dichlorophenolindophenol tests, along with blood indices, such as mean corpuscular volume and mean corpuscular hemoglobin, have limitations. Digital microscopy of peripheral blood smears is a promising alternative for objective quantification and standardization.

**Methods:**

Blood samples from 81 thalassemia screening‐negative and 41 screening‐positive individuals were analyzed using Mindray MC‐80 Digital Morphology.

**Results:**

Pre‐classification of red blood cell (RBC) morphology using Mindray MC‐80 revealed significant differences between the screening‐positive and screening‐negative groups. Various RBC morphologies demonstrated statistically significant variance, including hypochromic cells, schistocytes, elliptocytes, target cells, teardrop cells (*p* < 0.001), and ovalocytes (*p* = 0.002). However, the area under the receiver operating characteristic curve of these parameters was < 0.8, indicating a limited discriminatory power.

**Conclusion:**

RBC morphology showed promise in detecting subtle changes associated with thalassemia. However, it may not be sufficient for accurate screening alone, highlighting the need for complementary diagnostic approaches.

## Graphical abstract

RBC pre‐classification by MC‐80 has shown potential in identifying minor changes linked to thalassemia, but it may not be adequate for precise screening on its own.

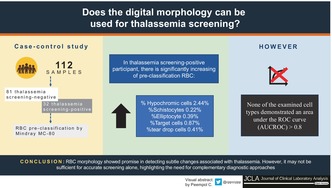

## 1 Introduction

Thalassemia is a genetic blood disorder affecting millions globally and is characterized by its autosomal recessive inheritance [1, 2]. Thalassemia is classified into alpha‐thalassemia and beta‐thalassemia based on which globin chain is affected. Alpha‐thalassemia results from deletions or mutations in the alpha‐globin genes, leading to a deficiency in alpha‐globin chain production, resulting in microcytic, hypochromic red blood cells (RBCs), target cells, and occasionally Heinz bodies, with clinical severity varying from mild anemia to more severe forms depending on the number of gene deletions. Beta‐thalassemia arises from mutations in the beta‐globin gene, causing reduced or absent beta‐globin chain production, leading to microcytosis, hypochromia, anisopoikilocytosis, and the presence of target cells and nucleated RBCs, and can present as thalassemia minor, intermedia, or major with varying degrees of clinical severity [3, 4].

Carriers of thalassemia may not show symptoms but can significantly affect their offspring, highlighting the importance of screening for pregnancy planning and reducing the associated morbidity and mortality [5, 6]. In Thailand, it is mandatory for all pregnant women and their partners to undergo thalassemia screening due to the risk of passing this genetic disorder to their offspring, potentially causing severe thalassemia. This regulation ensures early detection and management, making it an essential part of routine prenatal care [6]. Among the common diagnostic tools are blood indices, including mean corpuscular volume (MCV) and mean corpuscular hemoglobin (MCH), which are typically lower in thalassemic RBCs than in normal RBCs. The osmotic fragility (OF) test based on the heightened fragility of thalassemic RBCs and a dichlorophenolindophenol (DCIP) test, which detects abnormal hemoglobins like hemoglobin E (Hb E), were also utilized [6, 7, 8]. However, these methods have several limitations, including subjective interpretation and time‐consuming procedures [9].

Digital microscopy (DM) of peripheral blood smears (PBS) offers a promising alternative that provides objective quantification, standardization, and easier storage and sharing. Initially verified for leukocyte differentiation, DM has shown strong correlations between automated and manual evaluations across various cell types in the peripheral blood smears [10, 11]. Mindray MC‐80 (Mindray Bio‐Medical Electronics, Shenzhen, China) is an automated digital cell morphology analyzer that provides a percentage breakdown of each abnormal morphology with high throughput. Pre‐classification refers to the DM's initial classification and post‐classification for any manual adjustments made after that pre‐classification. Abnormal RBCs are commonly present in thalassemia or hemoglobin E carriers, including microcytes, hypochromia, or target cells [12]. While the MC‐80 can classify RBCs into microcyte, hypochromia, target cells, or other cells into the percentage of RBCs.

This study aimed to assess the diagnostic accuracy of digital imaging of PBS compared to traditional thalassemia screening tests, potentially offering valuable insights into the feasibility and efficacy of digital imaging and other diagnostic methods in thalassemia screening programs.

## 2 Materials and Method

Participants included in the study were individuals who had undergone routine thalassemia screening as part of antenatal care at the Hematology Laboratory of Songklanagarind Hospital, Prince of Songkla University, from June to December 2023. Our study included healthy pregnant women and their partners, who underwent thalassemia screening as part of routine prenatal care to identify carriers and assess the risk to their offspring. Blood samples were conveniently collected with ethylenediaminetetraacetic acid (EDTA) as an anticoagulant from routine samples sent for thalassemia screening.

All samples were tested for complete blood count (CBC) using a Mindray BC‐6200 (Mindray Bio‐Medical Electronics, Shenzhen, China). Samples with an MCV < 80 fL were considered positive. The OF test involved incubating the sample with 0.36% NaCl at room temperature for 5 min, followed by centrifugation and interpretation using an optical density (OD) reader. Samples with OD > 85 were considered positive. The DCIP test required incubating a sample of packed red cells with DCIP reagent at 37°C for 45 min before evaluation by manual visualization. Samples that presented precipitation were considered positive. A case was considered positive if it tested positive in any one of these tests (OF, DCIP, or MCV). PBS was automatically prepared and stained with May–Grünwald Giemsa using a Mindray SC‐120 Slidemaker/Stainer (Mindray Bio‐Medical Electronics, Shenzhen, China). Peripheral blood smears were automatically prepared and prefixed with methanol for 3 s, followed by air drying for 3 s. The smears were then stained with Wright‐Giemsa A solution (Baso Biotech, New Taipei City, Taiwan) for 1 min. Following this, the stain was diluted 1:10 with Wright‐Giemsa B buffer (Baso Biotech, New Taipei City, Taiwan) and allowed to stain for an additional 2 min. The digital morphology analysis of red blood cells (RBCs) was performed using the Mindray MC‐80 analyzer. The Mindray MC‐80 incorporates advanced microscopy features including high‐quality objectives, magnification up to 100×, automatic application of microscope oil, and an advanced sensor image camera. According to the manufacturer, the system captures images of the blood film and processes them at 20 different focal depths. It uses multi‐layer fusion technology to combine images obtained at different depths, reproducing cellular details as if visually focused under a microscope. RBC morphology was automatically determined by DM using a Mindray MC‐80, which is programmed to characterize and classify individual RBCs into the following cell classes: macrocytes, microcytes, hypochromic cells, polychromasia, schistocytes, echinocytes, elliptocytes, ovalocytes, stomatocytes, target cells, teardrop cells, and normal morphology RBCs.

Due to the absence of previous studies on digital morphology in thalassemia trait, we adhered to the Clinical and Laboratory Standards Institute EP‐09 guidelines for method comparison [13], which recommend a minimum of 40 samples per group to ensure reliable comparison. Our study included 85 screening‐negative and 41 screening‐positive participants, meeting these guidelines.

Positive screening in routine testing (OF, DCIP, or MCV) samples was performed for Hb typing using a Sebia CAPILLARYS 2 Flex Piercing analyzer (Sebia Cap 2FP; Sebia, Lisses, France). Samples with Hb A2 > 3.5% were considered to have beta‐thalassemia trait, while samples that contained Hb A and Hb E were considered to have beta^E^‐thalassemia trait.

For the statistical analysis, data were analyzed using descriptive statistics, including means and standard deviations or frequencies and percentages as appropriate. The results of the screening groups were compared using the Wilcoxon rank sum test or Fisher's exact test, depending on the data type.

Receiver operating characteristic (ROC) analysis was used to evaluate the discriminatory power between the positive and negative groups, with optimal cut‐off values determined by the point closest to (0,1) on the ROC Curve. All statistical analyses were performed using the epiDisplay (version 3.5.0.2) [14], tidyverse (version 1.3.1) [15], gtsummary (version 1.7.2) [16], and pROC (version 1.18.5) [17] packages in R language and environment version 4.2.1, as outlined by the R Core Team (2022). Results were deemed statistically significant at *p*‐values < 0.05.

This study was approved by the Human Research Ethics Committee of the Faculty of Medicine of Prince Songkla University, Thailand (REC: 66‐2305‐1). All samples were anonymized prior to analysis to protect participants' confidentiality and privacy. The study posed no additional risk to participants, as all analyses were performed on de‐identified samples, and the results did not affect patient care.

## 3 Result

Over the 6‐month period, 126 samples were eligible for our study. Baseline characteristics and hematological parameters are shown in Table 1 for 85 screening‐negative and 41 screening‐positive participants. Hb and hematocrit levels were significantly lower in the screening‐positive group than in the screening‐negative group. RBC counts were slightly higher in the screening‐positive group than in the screening‐negative group; however, this difference was not statistically significant. Significant differences were also observed in MCV and MCH, with screening‐positive participants showing lower values than screening‐negative participants, reflecting their use as discriminators. WBC and platelet counts did not differ significantly between the groups. Additionally, there were no significant differences in sex, age, and pregnancy status between the two groups.

**TABLE 1:** Baseline characteristics.

	Negative for thalassemia‐screening participants^a^	Positive for thalassemia‐ screening participants^a^	*p* ^b^
N	85	41	
Sex: Female	65 (76%)	33 (80%)	0.6
Age	34 (6)	32 (4)	0.4
Pregnancy status: Yes	48 (56%)	28 (68%)	0.2
Hb (g/dL)	13.35 (1.34)	12.21 (1.72)	< 0.001
RBC (*10^12^/L)	4.53 (0.46)	4.73 (0.66)	0.11
Hct (%)	40.0 (4.2)	36.8 (4.9)	< 0.001
MCV (pg/cell)	88.4 (4.6)	78.2 (6.7)	< 0.001
MCH (g/L)	29.51 (1.40)	25.95 (2.62)	< 0.001
WBC (*10^9^/L)	7.92 (2.24)	8.30 (1.75)	0.4
Platelet count (*10^9^/L)	287 (73)	290 (93)	0.8

*Note:* Continuous variables are represented as mean (SD), while dichotomous variables are represented as number (%).

Abbreviations: Hb, hemoglobin; Hct, hematocrit; MCH, mean corpuscular hemoglobin; MCV, mean corpuscular volume; RBC, red blood cell count; WBC, white blood cell count.

^a^ Positive for thalassemia‐ screening participants defined by positive either osmotic fragility, dichlorophenolindophenol test, or MCV lower than 80 fL.

^b^ Wilcoxon rank sum test.

A detailed analysis of the 41 screening‐positive participants revealed varied outcomes: low MCV in five individuals, positive DCIP test results in two, positive OF test results in eight, both low MCV and positive DCIP in five, low MCV and positive OF in six, and positivity across all screening assays in 11 cases.

**FIGURE 1:**
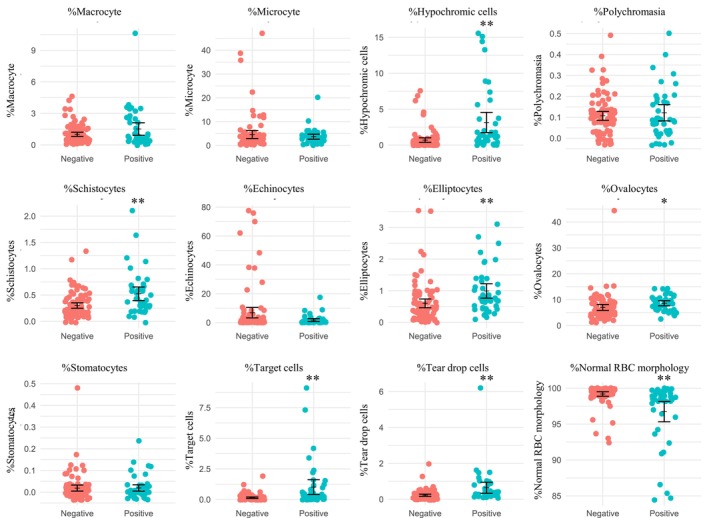
Percentage of pre‐classification red blood cell morphology by MC‐80 comparing negative‐thalassemia‐screening and positive thalassemia‐screening, Positive for thalassemia‐screening participants defined by positive either osmotic fragility, dichlorophenolindophenol test, or MCV lower than 80 fL. *p = 0.002, **p < 0.001.

In RBC pre‐classification using the MC‐80, significant morphological differences were noted between the screening‐positive and screening‐negative groups. Distinct cell types, including hypochromic cells, schistocytes, elliptocytes, ovalocytes, target cells, tear drop cells, and RBCs with normal morphology, demonstrated statistically significant variances, with *p*‐values < 0.001 for all except for ovalocytes, which had a p‐value of 0.002. An overview of the morphological differences between the positive and negative groups is presented in Figure 1 and Table 2.

**TABLE 2:** Percentage (SD) of different pre‐classification RBCs by MC80 for the negative and positive thalassemia screening test.

Variable	Negative for thalassemia‐ screening participants^a^ (*N* = 85)	Positive for thalassemia‐ screening participants^a^ (*N* = 41)	*p* ^b^
%Macrocytes	1.01 (0.93)	1.51 (1.88)	0.4
%Microcytes	4.6 (7.9)	3.7 (3.4)	0.2
%Hypochromic cells	0.70 (1.43)	3.14 (4.47)	< 0.001
%Polychromasia	0.11 (0.10)	0.12 (0.12)	0.7
%Schistocytes	0.30 (0.24)	0.52 (0.41)	< 0.001
%Echinocytes	7 (17)	2 (3)	0.2
%Elliptocytes	0.60 (0.65)	0.99 (0.72)	< 0.001
%Ovalocytes	7.0 (5.2)	8.6 (3.1)	0.002
%Stomatocytes	0.02 (0.06)	0.02 (0.05)	0.6
%Target cells	0.15 (0.27)	1.02 (1.90)	< 0.001
%Teardrop cells	0.23 (0.28)	0.64 (0.98)	< 0.001
%Normal Morphology RBC	99.19 (1.43)	96.73 (4.48)	< 0.001

*Note:* Continuous variables are represented as mean (SD).

Abbreviations: RBC, red blood cells.

^a^ Positive for thalassemia‐screening participants defined by positive either osmotic fragility, dichlorophenolindophenol test, or MCV lower than 80 fL.

^b^ Wilcoxon rank sum test.

**FIGURE 2:**
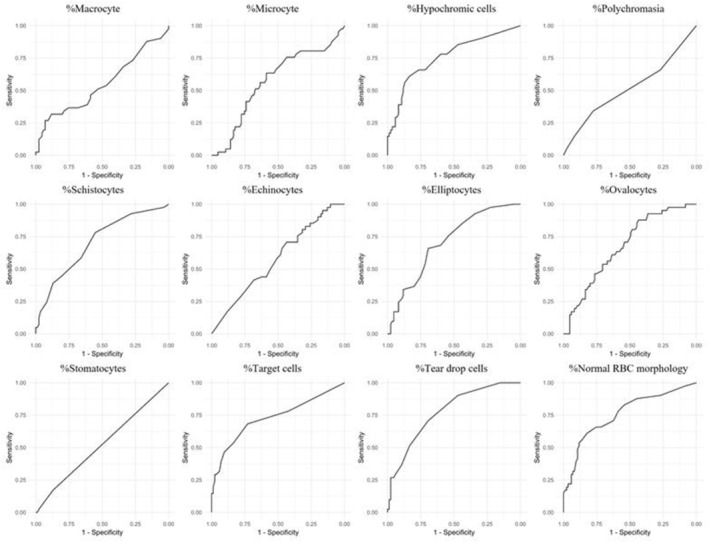
ROC curves each cell type from red blood cells pre‐classification using the MC‐80 to separate between negative‐thalassemia‐screening and positive thalassemia‐screening, Positive for thalassemia‐screening participants defined by positive either osmotic fragility, dichlorophenolindophenol test, or MCV lower than 80 fL.

In the ROC analysis, none of the examined cell types demonstrated an area under the ROC curve (AUCROC) < 0.8, indicating a lack of strong discriminatory power between the positive and negative groups based on ROC analysis. Among the variables, the highest AUC was observed for %teardrop cells, with an AUC of 0.77, suggesting it had the best performance in distinguishing between screening‐positive and screening‐negative groups, although still below the threshold for high diagnostic accuracy. The results and graphs of the ROC are shown in Figure 2 and Table 3.

**TABLE 3:** Area under the curve (AUC) from ROC curves (AUCROC) with optimal cut‐off values (%).

Variable	AUC	Optimal cut‐off value (%)^a^	Sensitivity	Specificity
%Macrocytes	0.55	0.85	0.46	0.59
%Microcytes	0.57	2.65	0.63	0.59
%Hypochromic cells	0.76	0.75	0.66	0.76
%Polychromasia	0.52	0.15	0.34	0.78
%Schistocytes	0.70	0.25	0.78	0.55
%Echinocytes	0.58	0.90	0.66	0.47
%Elliptocytes	0.70	0.65	0.66	0.69
%Ovalocytes	0.67	7.60	0.61	0.64
%Stomatocytes	0.52	0.05	0.17	0.87
%Targetcells	0.73	0.15	0.68	0.73
%Teardropcells	0.77	0.25	0.71	0.69
%Normal Morphology RBC	0.76	99.15	0.66	0.75

Abbreviations: AUC, area under the curve. RBC, red blood cells.

^a^ Optimal cut‐off values determined by the Point Closest to (0,1) on the ROC Curve.

All 41 patients who tested positive at the initial screening underwent Hb typing. One case was identified as beta‐thalassemia trait, 16 were classified as beta E‐thalassemia trait, and the remaining cases exhibited normal hemoglobin typing, although the alpha‐thalassemia trait could not be ruled out. Details of the screening test and RBC pre‐classification are listed in Table 4.

**TABLE 4:** Thalassemia screening test and RBC pre‐classification by MC80 of screening‐positive cases.

Variable	N	Normal hemoglobin typing cannot rule out alpha‐thalassemia trait	Beta‐thalassemia trait	Beta^E^‐thalassemia trait	*p*
Positive OF testing (*n* (%))	41	13 (54%)	1 (100%)	11 (69%)	0.7
Positive DCIP testing (*n* (%))	41	2 (8.3%)	0 (0%)	16 (100%)	< 0.001
MCV	41	81 (6)	62 (NA)	75 (5)	0.003
%Macrocytes	41	1.87 (2.25)	0.40 (NA)	1.04 (1.07)	0.4
%Microcytes	41	3.06 (2.34)	20.20 (NA)	3.64 (1.74)	0.13
%Hypochromic cells	41	3.4 (4.4)	1.2 (NA)	2.9 (4.7)	0.8
%Polychromasia	41	0.12 (0.13)	0.40 (NA)	0.11 (0.10)	0.2
%Schistocytes	41	0.52 (0.30)	2.10 (NA)	0.43 (0.36)	0.089
%Echinocytes	41	2.48 (4.11)	0.20 (NA)	0.69 (1.05)	0.2
%Elliptocytes	41	1.14 (0.68)	3.10 (NA)	0.64 (0.46)	0.008
%Ovalocytes	41	9.7 (2.9)	10.1 (NA)	6.8 (2.7)	0.007
%Stomatocytes	41	0.01 (0.03)	0.00 (NA)	0.03 (0.06)	0.5
%Targetcells	41	0.84 (1.91)	1.20 (NA)	1.28 (1.99)	0.4
%Teardropcells	41	0.44 (0.40)	6.20 (NA)	0.61 (0.41)	0.059
%Normal Morphology RBC	41	96.5 (4.4)	98.4 (NA)	97.0 (4.8)	0.8

*Note:* Continuous variables are represented as mean ± SD, while dichotomous variables are represented as number (%).

Abbreviations: DCIP, dichlorophenolindophenol test; MCV, mean corpuscular volume; OF, osmotic fragility; RBC, red blood cells.

## 4 Discussion

Our study evaluated baseline hematologic parameters in 85 thalassemia screening‐negative and 41 screening‐positive individuals. Morphological analysis through MC‐80 pre‐classification highlighted pronounced differences in specific RBC types, such as hypochromic cells, schistocytes, elliptocytes, ovalocytes, target cells, and teardrop cells, all exhibiting significant disparities except for ovalocytes. Despite these morphological variances, ROC analysis indicated that none of the cell types achieved an AUCROC > 0.8, suggesting limited efficacy in distinguishing between positive and negative screening groups based solely on these parameters. This highlights the nuanced nature of employing individual hematological parameters for screening objectives within our study context.

In this study, we explored the incorporation of digital morphology analysis of RBCs with traditional screening methods for identifying thalassemia carriers. Although traditional evaluation of RBC morphology is diagnostically valuable for numerous conditions, its subjectivity is a limiting factor [18]. The emergence of digital morphology analysis reflects trends toward objectivity and precision in the field. The OF, MCV, MCH, and DCIP tests have been used to screen for thalassemia carriers [19]. The OF test assesses the ability of RBCs to swell and lyse when exposed to solutions of decreasing osmolarity. Thalassemia carriers often have RBCs that are smaller and more resilient than normal, thus reducing their susceptibility to hemolysis [19, 20]. In this study, we used a single tube estimation at 0.36% NaCl for the OF, However, to avoid human error associated with visual interpretation, we employed spectrophotometric reading to assess the results. MCV and MCH were measured electronically using automated hematology analyzers, reflecting the size and Hb content of RBCs. As previously described, carriers may have smaller RBCs with lower Hb content than normal individuals [19]. Our study found that using DM showed a significantly lower percentage of hypochromic cells in the screening‐positive group, while the percentage of microcytic cells was slightly lower but not statistically significant. These findings confirm the lower Hb content of RBC in carriers is a characteristic that is reliably detectable using DM. Moreover, our study observed that RBC counts were slightly higher in the thalassemia screening‐positive, although this difference was not statistically significant. This can be attributed to the pathophysiology of thalassemia. Ineffective erythropoiesis and increased hemolysis in thalassemia lead to a compensatory increase in erythropoiesis, resulting in hypochromic microcytic red blood cells [21, 22].

The DCIP test has been used to screen an unstable Hb such as Hb E (β variant that can cause severe thalassemia) [23]. Regarding morphology, the number of target cells may be increased in individuals with Hb E in the RBCs [12]. In our study, DCIP testing demonstrates a correlation between the target cell percentage and the screening‐positive population. However, using the DCIP test to screen for Hb E can yield false positives, as observed in two cases. This could be due to the subjective nature of the test or the presence of other unstable Hb variants, such as the Hb Constant Spring. This variant is unstable, often present at low percentages, and is potentially degraded before testing [24].

No study has specifically targeted the minor morphological changes in thalassemia carriers. Common abnormalities in the RBCs of patients with thalassemia include target cells, ovalocytes, teardrop cells, stomatocytes, elliptocytes, and basophilic stippling [25]. Our study observed a significant increase in the percentages of target cells, ovalocytes, teardrop cells, schistocytes, and elliptocytes, indicating that carriers might exhibit abnormalities similar to those with the disease.

Huisjes et al. investigated the application of the CellaVision DM96 as a diagnostic aid for hereditary hemolytic anemia [26]. Their findings demonstrated that DM could effectively distinguish between various RBC disorders. In contrast, our study used DM to differentiate between screening‐positive for thalassemia trait and screening‐negative groups. Our results indicate that subtle changes in RBC morphology were insufficient to discriminate between these groups, as evidenced by the fact that the AUCROC for all cell types was < 0.8. This suggests that although RBC morphology can provide valuable insights into cellular abnormalities, detailed RBC morphology analysis alone does not provide adequate diagnostic, or screening accuracy for thalassemia.

Our study observed a higher variation in the percentage of echinocytes in thalassemia screening‐negative individuals, although this difference was not statistically significant. One possible explanation is the artificial formation of echinocytes due to the use of EDTA as an anticoagulant which can change RBC morphology to echinocyte during sample storage and preparation. This artifact formation could be more happened in the screening‐negative group, contributing to the higher variation observed.

One significant diagnostic challenge is the differentiation between thalassemia trait and iron deficiency anemia (IDA) due to overlapping laboratory findings such as microcytosis and hypochromia, which result in similar RBC indices. Thalassemia trait involves a genetic defect leading to abnormal hemoglobin production, whereas IDA results from a deficiency of iron necessary for hemoglobin synthesis. Although MCV and MCH are commonly used for screening, these indices can be reduced in both conditions, complicating the diagnosis. To address this, an iron profile—including serum ferritin, serum iron, total iron‐binding capacity (TIBC), and transferrin saturation—is crucial for a comprehensive assessment of iron status. Low serum ferritin and transferrin saturation indicate IDA, while normal or elevated ferritin levels with microcytosis suggest thalassemia trait [27, 28]. Our study did not include an iron profile in the screening process, which is a limitation. Future studies should incorporate iron profile testing to improve diagnostic accuracy and better differentiate between thalassemia trait and IDA, ensuring appropriate management and treatment.

Our study has several limitations. Firstly, the sample size of 126 individuals is relatively small for comprehensive thalassemia screening studies. Due to limitations in case availability and resource constraints, we were unable to increase the sample size. This small sample size may affect the generalizability of our findings. Despite this limitation, our study provides preliminary data on the feasibility and potential of using digital microscopy for thalassemia screening. Future studies with larger and more diverse populations are needed to confirm our findings and further evaluate the diagnostic accuracy of this approach. Secondly, our study relied on traditional screening tests, including MCV, OF test, and DCIP test, to identify thalassemia carriers. While these methods are useful for initial screening, they lack the specificity and sensitivity of high‐performance liquid chromatography, capillary electrophoresis, or molecular confirmation, which are the standard diagnostic methods for thalassemia [6, 7, 8]. Additionally, the DCIP test's reliance on manual visualization to detect hemoglobin precipitation introduces a level of subjectivity that can result in inconsistencies and potential inaccuracies. False positives may occur due to subjective interpretation, leading to the misclassification of individuals as carriers of unstable hemoglobin. The absence of molecular testing may result in false positives or false negatives, impacting the accuracy of our findings. For example, some individuals with borderline MCV values may be misclassified without genetic confirmation. Additionally, hematological indices, such as the Mentzer index, RDW index, and Shine & Lal index, have historically served as valuable tools in distinguishing thalassemia traits from iron deficiency anemia with a considerable degree of diagnostic accuracy. Considering the existing tools, introducing an additional screening method, particularly one with limited performance, appears redundant [29, 30]. Consequently, further research on DM for thalassemia screening appears unnecessary. Future studies should aim to integrate digital morphology with molecular testing for carrier status confirmation and explore the potential of artificial intelligence to improve diagnostic precision. Incorporating molecular confirmation will enhance the reliability of thalassemia screening and ensure that individuals are correctly identified as carriers or non‐carriers. Thirdly, while DM has advantages in analyzing RBC morphology, there may be technical limitations or performance variations across different platforms. The specific strengths and weaknesses of the Mindray MC‐80 system require further clarification. Additionally, there is no direct evidence regarding the efficacy of the Mindray MC‐80 in analyzing RBC morphology. While MC‐80's advanced imaging and software algorithms are anticipated to bring standardization and increased precision to RBC morphology evaluations, thereby aiding the diagnosis and management of hematologic conditions, the existing literature mainly addresses the overall performance of analogous technologies or different metrics, such as white blood cell differentials [31] and platelet counts [32], indicating a need for further research or detailed manufacturer documentation to fully understand the MC‐80's specific capabilities in RBC morphology analysis. Finally, the staining process can influence the morphology of RBCs [33], potentially affecting the accuracy of digital microscopic analysis. Variations in staining protocols could introduce inconsistencies in RBC morphology assessment. Standardizing staining procedures and ensuring consistent application across all samples is crucial. Future studies with larger samples should consider the influence of staining on RBC morphology and incorporate standardized staining protocols to minimize variability and improve the reliability of digital microscopy results.

In conclusion, our study used DM as a screening tool for analyzing RBC morphology during thalassemia screening. Various percentage differences in RBC morphology were observed between screening‐negative and screening‐positive patients. However, despite these findings, there are significant limitations to this study, including a small sample size, which would compromise the validity of our results and their extrapolation to the general population. Future studies should focus on combining digital morphology with molecular testing to confirm carrier status and consider exploring artificial intelligence to enhance diagnostic accuracy. While RBC morphology analysis alone cannot provide a definitive diagnosis, these integrated approaches can support and potentially enhance existing diagnostic tools in identifying thalassemia carriers accurately.

## Conflicts of Interest

The authors declare no conflicts of interest.

## Data Availability

The data that support the findings of this study are available on request from the corresponding author. The data are not publicly available due to privacy or ethical restrictions.

## References

[1] D. J. Weatherall and J. B. Clegg , “Inherited Haemoglobin Disorders: An Increasing Global Health Problem,” Bulletin of the World Health Organization 79, no. 8 (2001): 704–712. 11545326 PMC2566499

[2] N. J. Kassebaum , R. Jasrasaria , M. Naghavi , et al., “A Systematic Analysis of Global Anemia Burden From 1990 to 2010,” Blood 123, no. 5 (2014): 615–624. 24297872 10.1182/blood-2013-06-508325 PMC3907750

[3] F. B. Piel and D. J. Weatherall , “The α‐Thalassemias,” New England Journal of Medicine 371, no. 20 (2014): 1908–1916. 25390741 10.1056/NEJMra1404415

[4] A. T. Taher , K. M. Musallam , and M. D. Cappellini , “β‐Thalassemias,” New England Journal of Medicine 384, no. 8 (2021): 727–743. 33626255 10.1056/NEJMra2021838

[5] B. Modell , “Global Epidemiology of Haemoglobin Disorders and Derived Service Indicators,” Bulletin of the World Health Organization 2008, no. 6 (2008): 480–487. 10.2471/BLT.06.036673 PMC2647473 18568278

[6] S. Fucharoen , V. S. Tanphaichitr , K. Torcharus , V. Viprakasit , and A. Meekaewkunchorn , Thailand Clinical Practice Guidelines for Diagnosis and Management of Thalassemia Syndromes (Bangkok: Queen Sirirkit National Institute of Child Health, 2014).

[7] K. Prayongratana , C. Polprasert , K. Raungrongmorakot , K. Tatone , and S. Santiwatanakul , “Low Cost Combination of DCIP and MCV Was Better Than That of DCIP and OF in the Screening for Hemoglobin E,” Journal of the Medical Association of Thailand 91, no. 10 (2008): 1499–1504. 18972891

[8] O. Nathalang , K. Nillakupt , P. Arnutti , T. Boonsiri , S. Panichkul , and W. Areekul , “Screening for Thalassemia and Hemoglobinopathy in a Rural Area of Thailand: A Preliminary Study,” Journal of the Medical Association of Thailand 88, no. Suppl 3 (2005): S35–S42. 16862674

[9] T. Munkongdee , P. Chen , P. Winichagoon , S. Fucharoen , and K. Paiboonsukwong , “Update in Laboratory Diagnosis of Thalassemia,” Frontiers in Molecular Biosciences 7 (2020): 74. 32671092 10.3389/fmolb.2020.00074 PMC7326097

[10] H. Ceelie , R. B. Dinkelaar , and W. van Gelder , “Examination of Peripheral Blood Films Using Automated Microscopy; Evaluation of Diffmaster Octavia and Cellavision DM96,” Journal of Clinical Pathology 60, no. 1 (2007): 72–79. 16698955 10.1136/jcp.2005.035402 PMC1860603

[11] N. Khongjaroensakun , N. Chaothai , and P. Chinudomwong , “White Blood Cell Differentials Performance of a New Automated Digital Cell Morphology Analyzer: Mindray MC‐80,” International Journal of Laboratory Hematology 45, no. 5 (2023): 691–699. 37338111 10.1111/ijlh.14119

[12] S. Fucharoen and D. J. Weatherall , “The Hemoglobin E Thalassemias,” Cold Spring Harbor Perspectives in Medicine 2, no. 8 (2012): a011734. 22908199 10.1101/cshperspect.a011734 PMC3405827

[13] CLSI , Measurement Procedure Comparison and Bias Estimation Using Patient Samples; Approved, Third ed. CLSI document EP09‐A3. (Wayne, PA: Clinical and Laboratory Standards Institute, 2013).

[14] V. Chongsuvivatwong , epiDisplay: Epidemiological Data Display Package [Internet]. (Hat Yai, Thailand: Prince of Songkla University, 2022). https://CRAN.R‐project.org/package=epiDisplay.

[15] H. Wickham , M. Averick , J. Bryan , et al., “Welcome to the Tidyverse,” Journal of Open Source Software 4, no. 43 (2019): 16.

[16] D. D. Sjoberg , K. Whiting , M. Curry , J. A. Lavery , and J. Larmarange , “Reproducible Summary Tables With the Gtsummary Package,” R Journal 13, no. 1 (2021): 570.

[17] X. Robin , N. Turck , A. Hainard , et al., “pROC: An Open‐Source Package for R and S+ to Analyze and Compare ROC Curves,” BMC Bioinformatics 12, no. 1 (2011): 77. 21414208 10.1186/1471-2105-12-77 PMC3068975

[18] J. Ford , “Red Blood Cell Morphology,” International Journal of Laboratory Hematology 35, no. 3 (2013): 351–357. 23480230 10.1111/ijlh.12082

[19] V. Viprakasit and S. Ekwattanakit , “Clinical Classification, Screening and Diagnosis for Thalassemia,” Hematology/Oncology Clinics of North America 32, no. 2 (2018): 193–211. 29458726 10.1016/j.hoc.2017.11.006

[20] C. Kattamis , G. Efremov , and S. Pootrakul , “Effectiveness of One Tube Osmotic Fragility Screening in Detecting Beta‐Thalassaemia Trait,” Journal of Medical Genetics 18, no. 4 (1981): 266–270. 7277419 10.1136/jmg.18.4.266 PMC1048730

[21] A. Jassim , “Comparative Behavior of Red Blood Cells Indices in Iron Deficiency Anemia and β‐Thalassemia Trait,” Iraqi Journal of Hematology 5, no. 2 (2016): 183.

[22] A. Soliman , G. Kamal , W. Ae , and M. T. Sallam , “Blood Indices to Differentiate Between β‐Thalassemia Trait and Iron Deficiency Anemia in Adult Healthy Egyptian Blood Donors,” Egyptian Journal of Haematology 39, no. 3 (2014): 91.

[23] P. Kulapongs , T. Sanguansermsri , S. Tawarat , and G. Mertz , “Dichlorophenolindophenol (DCIP) Precipitation Test,” Journal of Associated Medical Sciences 9, no. 3 (2016): 161.

[24] M. F. Yusoff , M. TER , H. Hashim , and Z. Seman , “Evaluation of Dichlorophenolindophenol (DCIP) Test for Haemoglobin E (Hb E) in Normal Red Cell Indices Individuals,” Malaysian Journal of Medical Sciences 17 (2021): 10–13.

[25] C. Körber , A. Wölfler , M. Neubauer , and C. Robier , “Red Blood Cell Morphology in Patients With β‐Thalassemia Minor,” LaboratoriumsMedizin 41, no. 1 (2017): 49–52.

[26] T. Jameel , M. Baig , I. Ahmed , M. B. Hussain , and M. B. D. Alkhamaly , “Differentiation of Beta Thalassemia Trait From Iron Deficiency Anemia by Hematological Indices,” Pakistan Journal of Medical Sciences 33, no. 3 (2017): 665–669. 28811791 10.12669/pjms.333.12098 PMC5510123

[27] R. O. Wallerstein and P. M. Aggeler , “Anemia, Differentiating Between Thalassemia Minor and Iron Deficiency,” California Medicine 84, no. 3 (1956): 176–179. 13304673 PMC1532904

[28] R. Huisjes , W. W. van Solinge , M. D. Levin , R. van Wijk , and J. A. Riedl , “Digital Microscopy as a Screening Tool for the Diagnosis of Hereditary Hemolytic Anemia,” International Journal of Laboratory Hematology 40, no. 2 (2018): 159–168. 29090523 10.1111/ijlh.12758

[29] A. M. Maskoen , L. Reniarti , E. Sahiratmadja , J. Sisca , and S. H. Effendi , “Shine & Lal Index as a Predictor for Early Detection of β‐Thalassemia Carriers in a Limited Resource Area in Bandung, Indonesia,” BMC Medical Genetics 20, no. 1 (2019): 136. 31399060 10.1186/s12881-019-0868-x PMC6688316

[30] S. Tabassum , M. Khakwani , A. Fayyaz , and N. Taj , “Role of Mentzer Index for Differentiating Iron Deficiency Anemia and Beta Thalassemia Trait in Pregnant Women,” Pakistan Journal of Medical Sciences 38, no. 4 (2022): 878–882. 35634613 10.12669/pjms.38.4.4635 PMC9121960

[31] A. Merino , J. Laguna , M. Rodríguez‐García , J. Julian , A. Casanova , and A. Molina , “Performance of the New MC‐80 Automated Digital Cell Morphology Analyser in Detection of Normal and Abnormal Blood Cells: Comparison With the CellaVision DM9600,” International Journal of Laboratory Hematology 46, no. 1 (2024): 72–82. 37746889 10.1111/ijlh.14178

[32] Y. Üstündağ , K. Huysal , E. Güler Kazancı , F. Yıldırım , and M. R. Yeşil , “Use of Mindray MC‐80 Digital Morphology Analyzer's Estimated Platelet Counts as Adjunct to Automated Hematology Analyzer,” Acta Haematologica Polonica 54, no. 3 (2023): 169–175.

[33] R. V. Pierre , “Red Cell Morphology and the Peripheral Blood Film,” Clinics in Laboratory Medicine 22, no. 1 (2002): 25–61. 11933577 10.1016/s0272-2712(03)00066-0

